# Type-1 pericytes accumulate after tissue injury and produce collagen in an organ-dependent manner

**DOI:** 10.1186/scrt512

**Published:** 2014-11-06

**Authors:** Alexander Birbrair, Tan Zhang, Daniel Clark Files, Sandeep Mannava, Thomas Smith, Zhong-Min Wang, Maria Laura Messi, Akiva Mintz, Osvaldo Delbono

**Affiliations:** Department of Internal Medicine-Gerontology, Wake Forest School of Medicine, Medical Center Boulevard, Winston Salem, NC 27157 USA; Neuroscience Program, Wake Forest School of Medicine, Medical Center Boulevard, Winston Salem, NC 27157 USA; Department of Pulmonary, Wake Forest School of Medicine, Medical Center Boulevard, Winston Salem, NC 27157 USA; Department of Orthopedics, Wake Forest School of Medicine, Medical Center Boulevard, Winston Salem, NC 27157 USA; Department of Neurosurgery, Wake Forest School of Medicine, Medical Center Boulevard, Winston Salem, NC 27157 USA

## Abstract

**Introduction:**

Fibrosis, or scar formation, is a pathological condition characterized by excessive production and accumulation of collagen, loss of tissue architecture, and organ failure in response to uncontrolled wound healing. Several cellular populations have been implicated, including bone marrow-derived circulating fibrocytes, endothelial cells, resident fibroblasts, epithelial cells, and recently, perivascular cells called pericytes. We previously demonstrated pericyte functional heterogeneity in skeletal muscle. Whether pericyte subtypes are present in other tissues and whether a specific pericyte subset contributes to organ fibrosis are unknown.

**Methods:**

Here, we report the presence of two pericyte subtypes, type-1 (Nestin-GFP-/NG2-DsRed+) and type-2 (Nestin-GFP+/NG2-DsRed+), surrounding blood vessels in lungs, kidneys, heart, spinal cord, and brain. Using Nestin-GFP/NG2-DsRed transgenic mice, we induced pulmonary, renal, cardiac, spinal cord, and cortical injuries to investigate the contributions of pericyte subtypes to fibrous tissue formation *in vivo*.

**Results:**

A fraction of the lung’s collagen-producing cells corresponds to type-1 pericytes and kidney and heart pericytes do not produce collagen in pathological fibrosis. *Note that type-1, but not type-2, pericytes increase and accumulate near the fibrotic tissue in all organs analyzed.* Surprisingly, after CNS injury, type-1 pericytes differ from scar-forming PDGFRβ + cells.

**Conclusions:**

Pericyte subpopulations respond differentially to tissue injury, and the production of collagen by type-1 pericytes is organ-dependent. Characterization of the mechanisms underlying scar formation generates cellular targets for future anti-fibrotic therapeutics.

**Electronic supplementary material:**

The online version of this article (doi:10.1186/scrt512) contains supplementary material, which is available to authorized users.

## Introduction

The circulatory system supplies oxygen and nutrients to the entire organism. Cells tightly associated with the vasculature, called pericytes [1], stabilize the blood vessels in the microvasculature [2, 3], but recent studies suggest many other regulatory, immune, angiogenic, and phagocytic functions as well as a role in tissue homeostasis. Strong evidence indicates that pericytes are multipotent stem cells [4–17]. Besides their role in tissue repair, pericytes can trigger a fibrogenic response to pathological situations in some organs [18–22] but not others [23].

In tissue fibrosis, an integral component of most pathologic conditions, extracellular matrix synthesis is deregulated, leading to the destruction of organ architecture and impaired function [24]. The biological processes underlying fibrous tissue deposition are not fully understood. Besides pericytes, various cell types have been implicated: resident fibroblasts [25], bone marrow-derived circulating fibrocytes [26], epithelial cells [27], and endothelial cells [28].

Based on markers and morphology, pericytes are heterogeneous [29]. However, we were the first to demonstrate their diverse differentiation potential [30–33]. We identified two pericyte subtypes, type-1 and type-2 pericytes, using a double-transgenic Nestin-GFP/NG2-DsRed mouse. Under specific culture conditions, type-1 pericytes (Nestin-GFP^–^/NG2-DsRed^+^) generate adipocytes and fibroblasts but not neural cells, while type-2 pericytes (Nestin-GFP^+^/NG2-DsRed^+^) generate either Tuj1^+^ neural cells or become muscle cells [30, 31]. Recently, we showed that type-1 pericytes contribute to muscle fibrous tissue formation with aging [34]. Whether pericyte subtypes are present in other organs and whether their roles vary in tissue fibrogenesis are unknown. Reports indicate that pericytes may contribute to fibrosis in some organs [20, 22] but not others [23].

Here, using a Nestin-GFP/NG2-DsRed transgenic mouse, we distinguished the two pericyte subtypes associated with microvessels in the lung, kidney, heart, spinal cord, and brain. In addition, we used mouse models of pulmonary [21], renal [23], cardiac [35], and central nervous system (CNS) [36] fibrosis to investigate the contributions of pericyte subtypes to fibrous tissue formation *in vivo*. Because collagen type I production increases several hundred-fold in pathological fibrosis [37], we evaluated whether the two pericyte subtypes were among the collagen-producing cells in diseased organs. We found that type-1 pericytes but not type-2 pericytes produce collagen type I in bleomycin-induced pulmonary fibrosis, but they are only a fraction of the total collagen-producing cells; and although they increase and accumulate near the fibrotic tissue, they do not produce collagen type I in renal and cardiac fibrosis. Similarly, after CNS injury, type-1 pericytes participate in the scar tissue but differ from the PDGFRβ^+^ cells that are responsible for collagen production [36, 38]. Consequently, the exact role of type-1 pericytes in tissue fibrosis requires further research.

We conclude that the pericyte subtypes respond differentially to tissue injury; type-1 pericytes do or do not produce collagen depending on the organ injured, and scar formation mechanisms are specific to the injured organ. Further study of pericyte subtype functions in peripheral tissues may reveal ways to improve tissue repair under pathological conditions or with aging.

## Materials and methods

### Animals

Nestin-GFP transgenic mice were maintained homozygous for the transgene on the C57BL/6 genetic background [39, 40]. These mice were crossbred with NG2-DsRed transgenic mice expressing DsRed-T1 under the control of the NG2 promoter [41] (purchased from Jackson Laboratory, Bar Harbor, ME, USA) to generate Nestin-GFP/NG2-DsRed double-transgenic mice. All mouse colonies were housed at Wake Forest School of Medicine in a pathogen-free facility of the Animal Research Program under a 12-hour:12-hour light/dark cycle and fed *ad libitum*. Both male and female homozygous mice were used, and their ages ranged from 3 to 5 months. Animal handling and procedures were approved by the Wake Forest School of Medicine Animal Care and Use Committee.

### Mouse model of lung injury with bleomycin

For intratracheal instillation of bleomycin, Nestin-GFP/NG2-DsRed double-transgenic mice were anesthetized with an intraperitoneal injection of 114 mg/kg ketamine and 17 mg/kg xylazine, and the trachea was exposed. The animals were orotracheally intubated with an 18-gauge catheter; 0.025 units of pharmaceutical grade bleomycin in 50 μl H_2_O were delivered intratracheally. The incision was closed, and the catheter removed. Lungs were harvested at day 14.

For lung histology and immunohistochemistry, at the time of sacrifice, the lungs were perfused with 1 ml phosphate-buffered saline (PBS) through the right ventricle. The right lung was tied off with suture, removed, and frozen in liquid nitrogen. The left lung was perfused with 1 ml of 4% paraformaldehyde/PBS. The left lung was then inflated to 25 mmHg with 1% agarose/4%paraformaldehyde/PBS. The lung was tied off with a suture, removed, and placed in 4% paraformaldehyde overnight. The following day, the lung was placed in 5% sucrose/PBS for 6 hours at 4°C, then in 20% sucrose/PBS overnight. Next day, the lung was placed in a cryomold (TissueTek, Sakura Finetek, Torrance, CA, USA), covered in Optimal Cutting Tissue (TissueTek), and flash frozen in ice-cold isopentane [42, 43].

### Unilateral ureteral obstruction mouse model

A unilateral ureteral obstruction (UUO) kidney disease model was induced as described previously [44–47]. UUO or sham surgery was performed in Nestin-GFP/NG2-DsRed mice. All surgeries were performed under general anesthesia with isoflurane. In the UUO group, the right ureter was exposed through an abdominal midline incision and ligated using 4–0 silk under sterile conditions. Volume depletion was prevented by administering 0.1 ml saline into the peritoneal cavity. The midline incision was closed; the mice were returned to their cages and allowed free access to food and water. Sham-operated mice had their ureters exposed and manipulated without ligation. Sham (nonobstructed) kidneys and UUO (obstructed) kidneys were harvested at day 14 post surgery for tissue analysis.

### Mouse model of myocardial infarction

Myocardial infarction (MI) was induced by ligating the left anterior descending (LAD) coronary artery in aseptic conditions as described previously [48–50]. Briefly, Nestin-GFP/NG2-DsRed mice were anesthetized with an intraperitoneal injection of ketamine and xylazine (50 and 10 mg/kg, respectively). The mice were then intubated and connected to a ventilator (Kent Scientific model RSP1002 (Kent Scientific Corporation, Litchfield, CT, USA)) and anesthesia was maintained using isoflurane (1 to 1.5 vol% in oxygen). Mouse core temperature was monitored and maintained at 35 to 37°C using a thermal pad and heat lamp. The left wall of the thorax was shaved, cleansed with betadine scrub, and disinfected with betadine solution. A 1 cm incision was made perpendicular to the sternum at the sixth intercostal space. The muscles overlying the sixth intercostal space were transected and the intercostal muscles were cut, exposing the pericardial sac. A cotton swab was placed in the chest behind the heart to elevate the heart and rotate it medially. A 9–0 or 10–0 nylon monofilament suture was passed through the myocardium approximately 1 to 2 mm below the edge of the left atrium along a line extending from the junction of the ascending aorta and the pulmonary artery toward the apex of the heart. This suture was tied in place, constricting the coronary artery and causing visible blanching and dyskinesia of the left ventricle. The cotton swab was then removed and the lungs were inflated to remove any atelectasis. A silicone rubber chest tube was placed between the ribs at the site of the thoracotomy and subatmospheric pressure of 15 cmH_2_O was applied. The thoracotomy was closed with 6–0 coated vicryl. The overlying muscles were approximated using 6–0 coated vicryl, as was the skin. After the skin incision was closed, the chest tube was removed and the mouse was removed from the ventilator with the endotracheal tube in place until spontaneous breathing began and the mouse began to awaken from the anesthetic. The mouse was then extubated and monitored until he regained consciousness and movement about the cage.

After induced MI and as needed, animals received analgesics based on signs of distress (decreased activity, piloerection, ungroomed appearance, excessive licking and scratching, self-mutilation, abnormal stance, hunched appearance, rapid or shallow respiration, grunting, dilated pupils, aggression toward handler, high-pitched vocalizations, change in feeding activity, and attempts to separate from the group). Mice with clinical distress were euthanized. Mice were killed by cervical dislocation, and heart samples were harvested postmortem for histological analyses.

### Histology of the lung, kidney, and heart

Histological studies in the lung, kidney, and heart followed standard procedures. Lungs were collected 2 weeks after intratracheal bleomycin or saline administration; kidneys 14 days after UUO or sham surgery; and hearts 2 weeks after myocardial infarction or sham surgery. Tissues were fixed in 4% formalin in PBS, embedded in paraffin, and sectioned at 5 μm intervals to perform hematoxylin and eosin staining for general morphology and Van Gieson and Masson’s trichrome staining for collagen accumulation.

### Immunohistochemistry of the lung, kidney, and heart

To detect DsRed and GFP fluorescence or GFP fluorescence alone in Nestin-GFP/NG2-DsRed mice, the hearts, kidneys, and lungs were dissected, fixed in 4% paraformaldehyde overnight, immersed in 10%, 20%, and 30% sucrose solutions for 60, 45, and 30 minutes, respectively, embedded in Optimal Cutting Temperature compound (OCT), and rapidly frozen in liquid nitrogen to prepare cryosections 10 μm thick. These sections were permeabilized in 0.5% Triton X-100 (Sigma, St. Louis, MO, USA), and blocked to saturate nonspecific antigen sites using 5% (v/v) goat serum/PBS (Jackson Immunoresearch Labs, West Grove, PA, USA) overnight at 4°C. Next day, the sections were incubated with primary antibodies (anti-CD31 (PECAM-1 antibody; BD Biosciences, San Jose, CA, USA) at 1:100 dilution or anti-collagen type I antibody (AbD Serotec, Raleigh, NC, USA) at 1:1,000 dilution) at room temperature for 4 hours and visualized using appropriate species-specific secondary antibodies conjugated with Alexa Fluor 488, 568, 647, or 680 at 1:1,000 dilution (Invitrogen, Carlsbad, CA, USA). Tissue sections were counterstained with Hoechst 33342, a nuclear marker, were mounted on slides using Fluorescent Mounting Medium (DakoCytomation, Carpinteria, CA, USA), and were examined with fluorescence microscopy.

### Spinal cord injury

To evaluate the cellular composition of spinal cord scars, surgery was performed on Nestin-GFP/NG2-DsRed mice, anesthetized by intraperitoneal injection of a ketamine/xylazine mixture, using a surgical microscope. Mice lay prone and immobilized on a special operating table, and the fur on their backs was shaved. Following a midline skin incision between the thoracic and lumbar regions and paravertebral muscle dissection, the spinous processes and laminar arcs were removed. The spinal cord was exposed and the dorsal funiculus was cut transversely, short of the gray matter and central canal, and extended caudally with microsurgical scissors to span one segment. The razor was as sharp as possible to minimize traumatic injury. Finally, the muscle and incision were sutured. To prevent infection and alleviate pain and/or discomfort, all animals received antibiotic (gentamycin, 5.8 mg/kg) and an analgesic (buprenorphine, 0.1 to 0.5 mg/kg) and were monitored for body weight and ambulatory, feeding, and grooming behavior. Two weeks after injury, the mice were anesthetized again and transcardially perfused with cold PBS followed by perfusion with 4% paraformaldehyde solution in PBS. Following perfusion, the spinal cord was removed, immersed for 24 hours in fixative solution, and cryoprotected with 30% sucrose in PBS for 2 days. The spinal cords were then placed in embedding cryomolds, covered with tissue-embedding medium (Tissue-Tek O.C.T. compound; Sakura Finetek, Tokyo, Japan), snap-frozen in liquid nitrogen, and stored at -80°C.

### Spinal cord immunohistochemistry

Frozen spinal cords were cut longitudinally and transversely into serial sections 20 μm thick using a cryostat at -20°C. Mounted on slides in series of six (Fisher Scientific, Pittsburgh, PA, USA), they were stored at -20°C before processing for immunocytochemistry. Sections were dried at room temperature for 1 hour, rehydrated in PBS, permeabilized with 0.5% Triton X-100 (Sigma) in PBS solution, and blocked to saturate nonspecific antigen sites using 5% (v/v) goat serum/PBS (Jackson Immunoresearch Labs) at 4°C overnight. The next day, the sections were incubated with primary antibodies at room temperature for 4 hours and visualized using appropriate species-specific secondary antibodies. Hoechst 33342 was used as a nuclear marker.

### Brain injury

To evaluate cell composition in scars formed after lesion, Nestin-GFP/NG2-DsRed mice were anesthetized with ketamine/xylazine as described above and positioned in a Just For Mice stereotaxic apparatus (Harvard Apparatus, Holliston, MA, USA). To expose the cortex, an opening 1 mm wide was made in the skull 1.5 mm from the midline, extending from bregma to lambda. A sterile metallic surgical blade, size 15 (BD Biosciences), was lowered 1.5 mm (relative to the dura mater) unilaterally into the cortex and drawn along the length of the exposed brain, parallel to the midline. To prevent infection and alleviate pain and/or discomfort, all animals received antibiotic (gentamycin, 5.8 mg/kg) and an analgesic (buprenorphine, 0.1 to 0.5 mg/kg). Their body weight and ambulatory, feeding, and grooming activities were monitored. Two weeks after injury, they were anesthetized and transcardially perfused with cold PBS followed by perfusion with 4% paraformaldehyde solution in PBS. After decapitation, their brains were rapidly dissected out, removed from the skull, postfixed for 24 hours in the same fixative solution, and cryoprotected with 30% sucrose in PBS for 2 days. The brains were then placed in embedding cryomolds, covered with tissue-embedding medium, snap-frozen in liquid nitrogen, and stored at -80°C.

### Brain immunohistochemistry

Frozen brains from Nestin-GFP/NG2-DsRed mice with or without injury were sectioned transversely into serial coronal sections 20 μm thick using a cryostat (Microm HM 500; Zeiss, Oberkochen, Germany) at -20°C, mounted on SuperFrost Plus Microscope Slides (Fisher Scientific) in series of six, and stored at -20°C before processing for immunocytochemistry. Sections were dried at room temperature for 1 hour, rehydrated in PBS, permeabilized with 0.5% Triton X-100 (Sigma) in PBS solution, and blocked to saturate nonspecific antigen sites using 5% (v/v) goat serum/PBS (Jackson Immunoresearch Labs) at 4°C overnight. The next day, the sections were incubated with anti-PDGFRβ antibody (gift from Dr W Stallcup, Sanford-Burnham Medical Research Institute, La Jolla, CA, USA) at 1:100 dilution at room temperature for 4 hours and visualized using appropriate species-specific secondary antibodies. Hoechst 33342 was used to mark nuclei. The sections were mounted on slides using Fluorescent Mounting Medium (DakoCytomation) and examined with fluorescence microscopy [51].

### Microscopy, cell imaging, and counting

An inverted motorized fluorescent microscope (IX81; Olympus, Tokyo, Japan) with an Orca-R2 Hamamatsu CCD camera (Hamamatsu, Hamamatsu City, Japan) was used for image acquisition. Camera drive and acquisition were controlled by a MetaMorph Imaging System (Olympus, Center Valley, PA, USA) [52, 53]. Ten arbitrary microscopic fields were counted in each immunostained plate or tissue section, and values pooled from parallel duplicates per time point and individual experiment.

### Statistical analysis

Results are expressed as the mean ± standard error of the mean. Statistical significance was assessed using Student’s *t* test or analysis of variance with GraphPad Prism (GraphPad Software, San Diego, CA). *P* < 0.05 was considered significant.

## Results

### Type-1, but not type-2, pericytes produce collagen type I in response to lung injury

**Figure 1:**
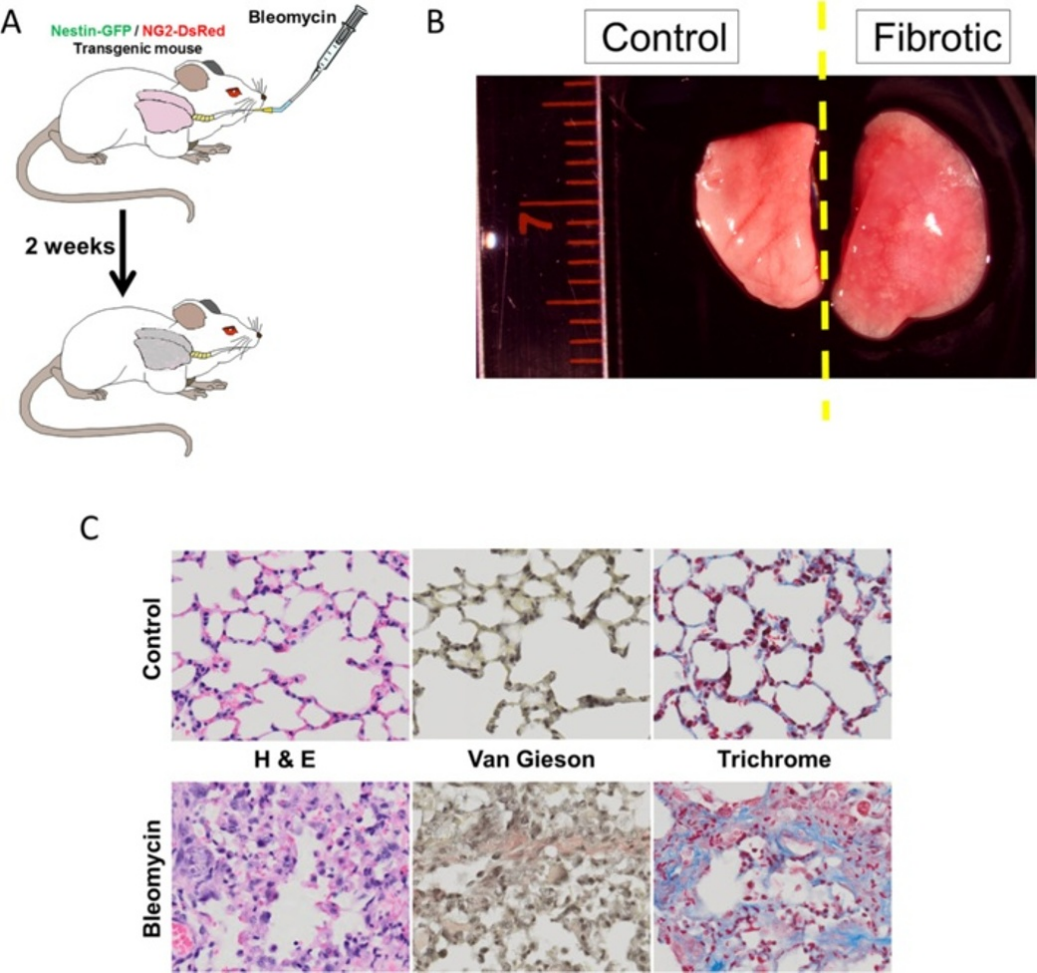
**Mouse model of lung injury with bleomycin. (A)** Schematic diagram of the experimental plan for inducing peribronchial fibrosis. **(B)** Gross anatomy representative of normal and fibrotic mouse lungs used in the study. Right lungs of Nestin-GFP/NG2-DsRed mice were used for classical histology **(C)**, and left lungs for immunohistochemistry (Figure 2A,B). **(C)** Representative sections of normal mouse lungs and fibrotic lungs collected 14 days after intratracheal administration of bleomycin. Images of sections stained with hematoxylin and eosin (H&E) for general morphology and van Gieson (pink) and Masson’s trichrome (blue) for collagen deposition throughout the lungs.

**Figure 2:**
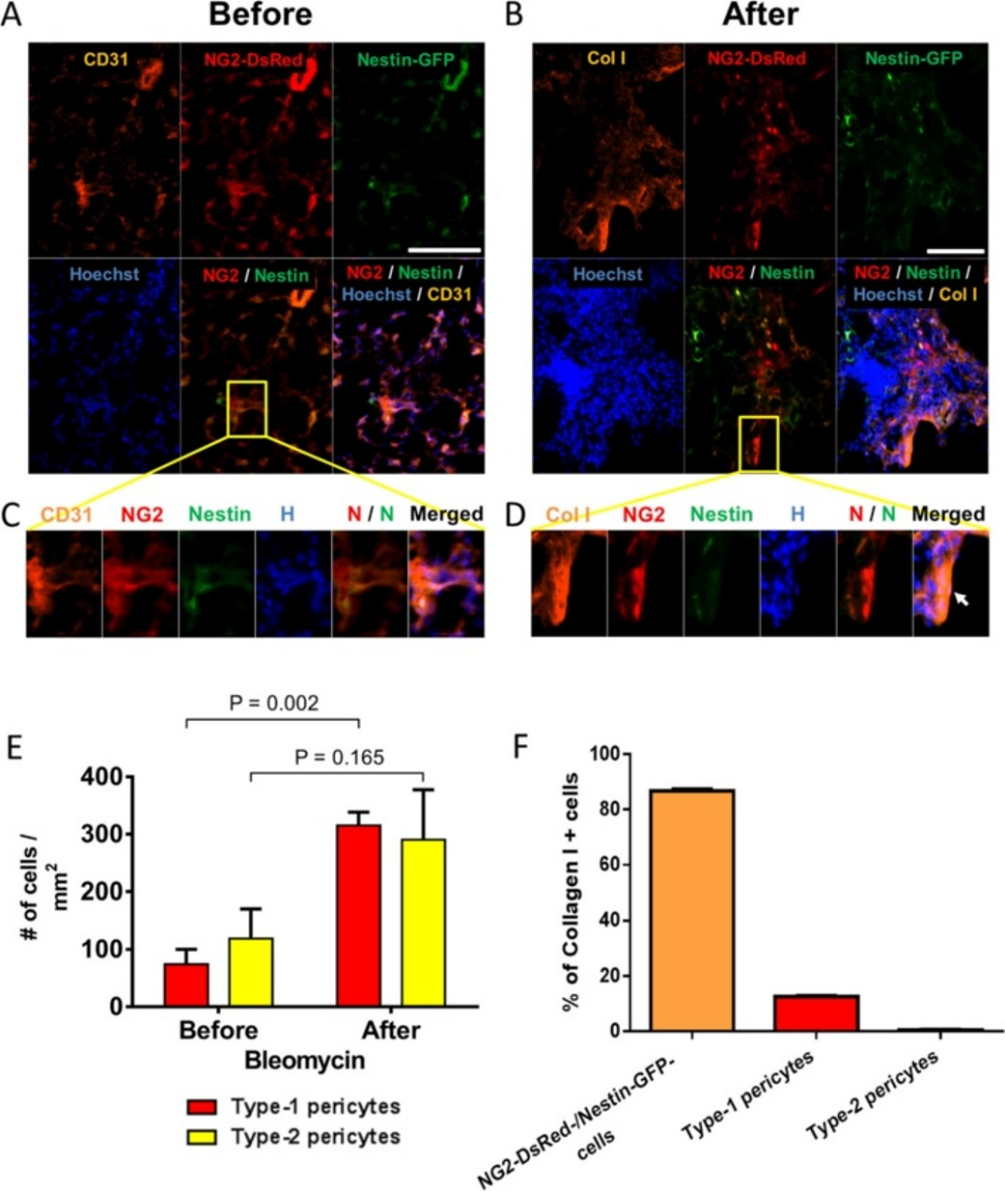
**Nestin-GFP** ^**–**^ **/NG2-DsRed** ^**+**^ **cells, but not Nestin-GFP** ^**+**^ **/NG2-DsRed** ^**+**^ **cells, increase and participate in pulmonary fibrosis. (A)** Representative photomicrographs of lung sections from Nestin-GFP/NG2-DsRed mice (control) showing blood vessels with CD31^+^ endothelial cells and pericytes (NG2-DsRed^+^). All panels show identical areas with CD31 staining, NG2-DsRed, Nestin-GFP^+^, Hoechst, and combined fluorescent images. Nestin-GFP^–^ and Nestin-GFP^+^ pericytes (NG2-DsRed^+^) surround capillaries. Region in yellow box shows type-1 and type-2 pericytes close to CD31^+^ blood vessels, magnified in (C). **(B)** Immunohistochemical staining with an antibody against type I collagen (Col I) in lung sections from Nestin-GFP/NG2-DsRed double-transgenic mice showing matrix deposition 2 weeks after bleomycin treatment. All panels show the same lung area with Col I, NG2-DsRed, Nestin-GFP^+^, Hoechst, and merged fluorescent images. Region in yellow box shows area with dense collagen accumulation at the lung injury site, magnified in (D). **(C)** Type-1 and type-2 pericytes close to CD31^+^ blood vessels, magnified from (A). **(D)** Dense collagen accumulation at the lung injury site, magnified from (B). Note that some type-1 pericytes (Nestin-GFP^–^/NG2-DsRed^+^) produce collagen type I after lung injury, indicated by a white arrow. **(E)** Number of type-1 and type-2 pericytes before and after pulmonary injury (*n* = 3 mice; 10 lung sections from each). **(F)** Percentage of cells producing type I collagen in NG2-DsRed^–^/Nestin-GFP^–^ cell populations, and type-1 and type-2 pericytes. Scale bars = 100 μm.

Several cell types contribute to fibrosis in response to bleomycin-induced lung injury [54, 55] (Figure 1A,B,C), including pericytes [21]. As the functions of the pericyte subtypes differ in skeletal muscle [31], we examined their role in pulmonary fibrosis in Nestin-GFP/NG2-DsRed mice. We detected type-1 and type-2 pericytes associated with lung CD31^+^ microvessels (Figure 2A,C) and treated Nestin-GFP/NG2-DsRed mice with the fibrogenic agent bleomycin [56] (Figure 1A,C). We looked for the pericyte subtypes in lung sections before and 2 weeks after the injury. We found that, compared with pre treatment (74 ± 26 cells/mm^2^), the number of type-1 pericytes (315 ± 23 cells/mm^2^) increased significantly after bleomycin treatment (*P* = 0.002). In contrast, the increase in type-2 pericytes was not significant (pre treatment, 119 ± 51 cells/mm^2^; post treatment, 290 ± 87 cells/mm^2^; *P* = 0.165) (Figure 2B,D,E). Moreover, type-1 pericytes correspond to 12.6 ± 0.3% of type I collagen-producing cells, while type-2 pericytes generated no detectable collagen (0.3 ± 0.3%) (*P* < 0.0001) (Figure 2B,D,F). As the connective tissue forming cells are denominated myofibroblasts [57], our results indicate that type-1 pericytes accumulate at the injury site and behave as such, contributing to pulmonary fibrosis.

### Type-1 pericytes accumulate in the kidney after unilateral ureteral obstruction

**Figure 3:**
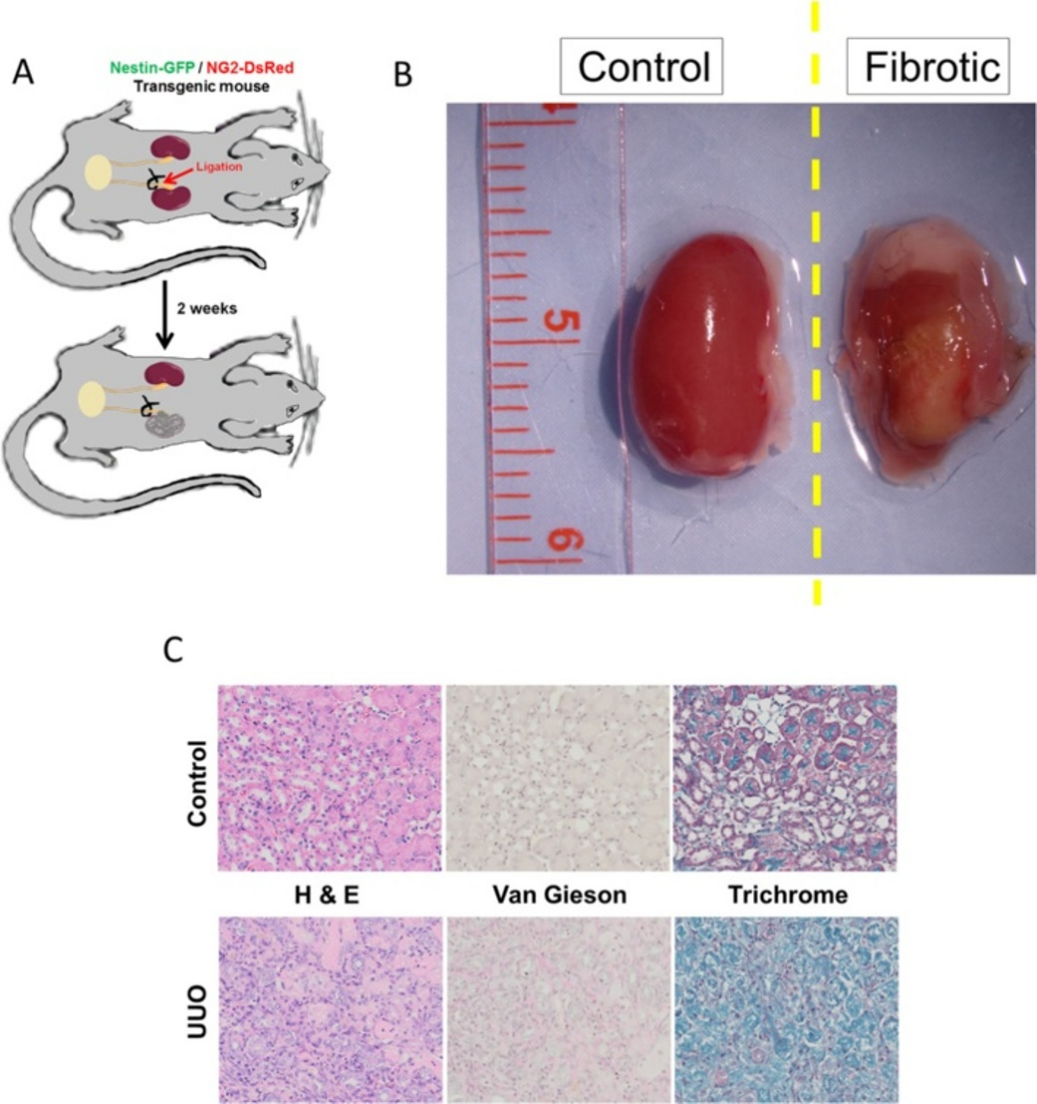
**Mouse model of unilateral ureteral obstruction. (A)** Schematic diagram showing unilateral ureteral obstruction (UUO) in Nestin-GFP/NG2-DsRed transgenic mice. The right ureter was exposed via a lateral incision and ligated. The right obstructed kidney or left nonobstructed kidney (control) was analyzed 14 days after the operation. **(B)** Gross anatomy representative of normal and fibrotic mouse kidneys used in the study. **(C)** Histology of UUO and contralateral kidneys. Paraffin kidney sections were stained with hematoxylin and eosin (H&E). Collagen content was assessed by van Gieson (pink) and Masson’s trichrome (blue).

**Figure 4:**
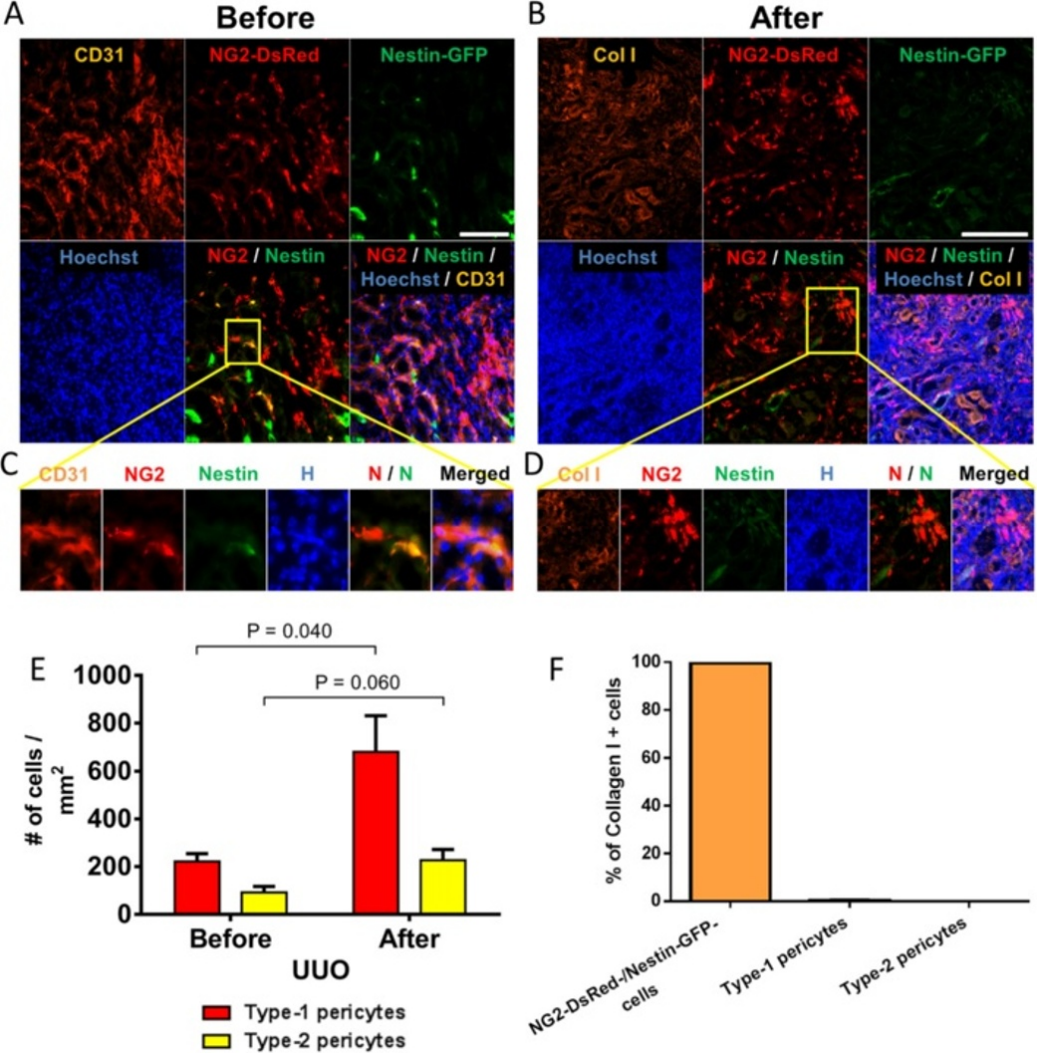
**NG2-DsRed** ^**+**^ **pericyte accumulation, but no collagen production, in a mouse model of kidney fibrosis. (A)** Immunohistochemistry of unobstructed kidney sections in a Nestin-GFP/NG2-DsRed mouse showing blood vessels labeled with the endothelial cell marker CD31; Nestin-GFP^–^-/NG2-DsRed^+^ (type-1) and Nestin-GFP^+^/NG2-DsRed^+^ (type-2) pericytes are attached to it. All panels show the same area for different channels (CD31, NG2-DsRed, Nestin-GFP, Hoechst, and merged images). Region in yellow box shows type-1 and type-2 pericytes close to CD31^+^ blood vessels, magnified in (C). **(B)** Representative immunofluorescence staining of type I collagen (Col I) in the kidney 14 days after UUO in a Nestin-GFP/NG2-DsRed mouse. All panels show the same area for different channels (Col I, NG2-DsRed, Nestin-GFP, Hoechst, and merged images). Region in yellow box shows an area with high type-1 pericyte concentration, magnified in (D). **(C)** Type-1 and type-2 pericytes close to CD31^+^ blood vessels, magnified from (A). **(D)** High type-1 pericyte concentration, magnified from (B). Note that in this model of kidney fibrosis, NG2-DsRed^+^ cells do not express Col I. **(E)** Quantification of type-1 and type-2 pericytes before and 14 days after UUO (*n* = 3 mice; 10 kidney sections from each). **(F)** Percent of cells expressing Col I in the kidney 2 weeks after UUO. Note that NG2^+^ pericytes do not contribute to collagen type I production. Scale bars = 100 μm.

UUO in rodents generates progressive renal fibrosis (Figure 3A,B,C). Although several studies have suggested that pericytes, among other cell types [58–62], contribute to collagen production in kidney fibrosis [63–65], a recent study ablated them and found no significant reduction in fibrosis but significant accumulation in the interstitium [23]. We detected type-1 and type-2 pericytes attached to renal CD31^+^ microvasculature (Figure 4A,C). To determine whether both pericyte subtypes accumulate after renal fibrosis, we induced UUO in Nestin-GFP/NG2-DsRed mice and evaluated fibrosis by hematoxylin and eosin, Van Gieson, and Masson’s trichrome staining (Figure 3A,B,C). Compared with the sham control, the UUO group showed much more collagen deposition and more prominent fibrosis at day 14 (Figure 3C). We observed a high concentration of type-1 pericytes near areas of dense collagen accumulation, but they were not producing type I collagen (Figure 4B,D,F; see Additional file 1). The number of type-1 pericytes increased significantly 2 weeks after UUO (pre injury, 222 ± 32 cells/mm^2^; post injury, 681 ± 150 cells/mm^2^; *P* = 0.040), with an insignificant increase in type-2 pericytes (*P* = 0.060) (Figure 4B,D,E).

### Type-1 pericytes accumulate and surround the fibrotic area after myocardial infarction

**Figure 5:**
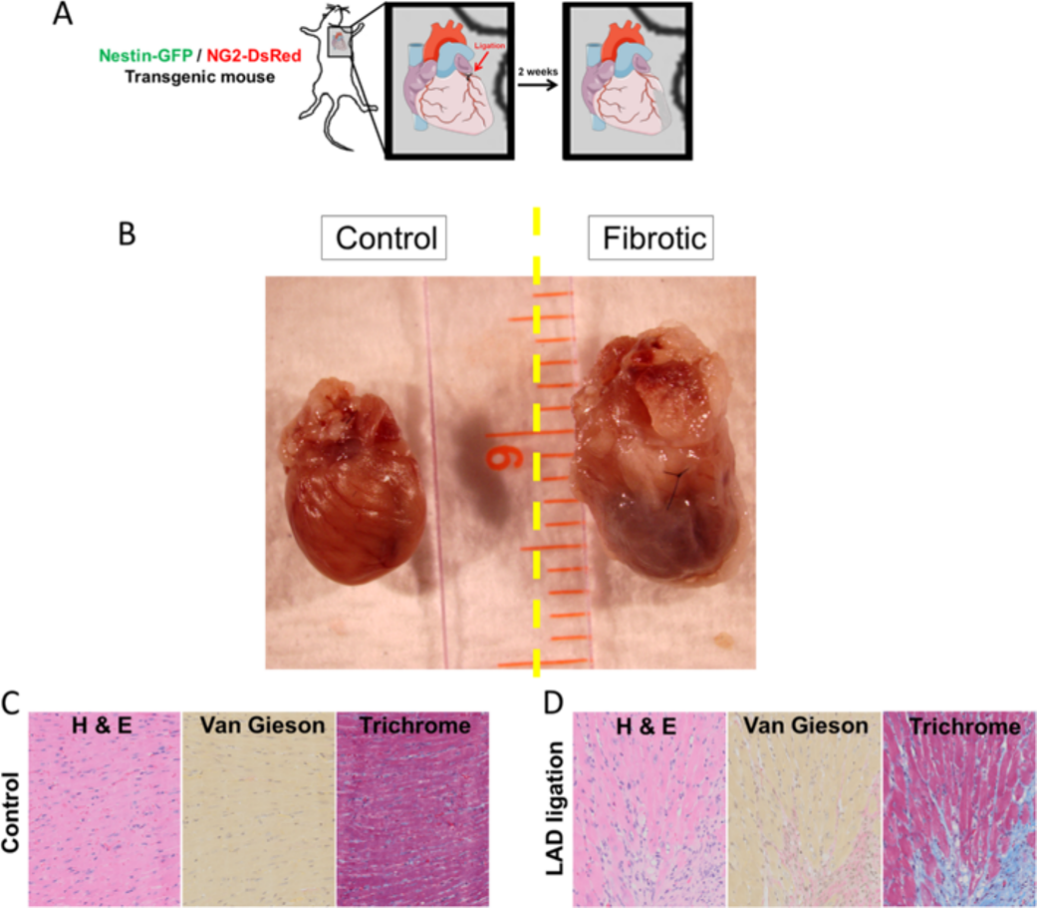
**Type-1 pericytes accumulate in the fibrotic region after myocardium infarction but do not express collagen. (A)** Representative photomicrographs of longitudinal sections of myocardial tissue from Nestin-GFP/NG2-DsRed double-transgenic mice. Blood vessels with CD31^+^ endothelial cells are surrounded by Nestin-GFP^–^/NG2-DsRed^+^ (type-1) and Nestin-GFP^+^/NG2-DsRed^+^ (type-2) pericytes. All panels show the same area for different channels (CD31, NG2-DsRed, Nestin-GFP, Hoechst, and merged fluorescence). Region in yellow box shows type-1 and type-2 pericytes close to CD31^+^ blood vessels, magnified in (C). **(B)** Representative longitudinal sections of hearts 14 days post infarct from Nestin-GFP/NG2-DsRed double-transgenic mice. All panels show identical areas in the heart section (CD31 staining, NG2-DsRed, Nestin-GFP^+^, Hoechst, and merged fluorescence images). Region in yellow box shows area with high type-1 pericyte accumulation near areas with dense collagen production, magnified in (D). **(C)** Type-1 and type-2 pericytes close to CD31^+^ blood vessels, magnified from (A). **(D)** High type-1 pericyte accumulation near areas with dense collagen production, magnified from (B). Note that anti-type I collagen (Col) staining confirms that neither type-1 nor type-2 pericytes express Col I, although type-1 pericytes accumulate near the fibrotic area. **(E)** Quantification of type-1 and type-2 pericytes before and 14 days after infarction (*n* = 3 mice; 10 heart sections from each). Note that the number of type-1 pericytes increased significantly. **(F)** Percent of cells expressing Col I in the infarcted heart. Scale bars = 100 μm.

**Figure 6:**
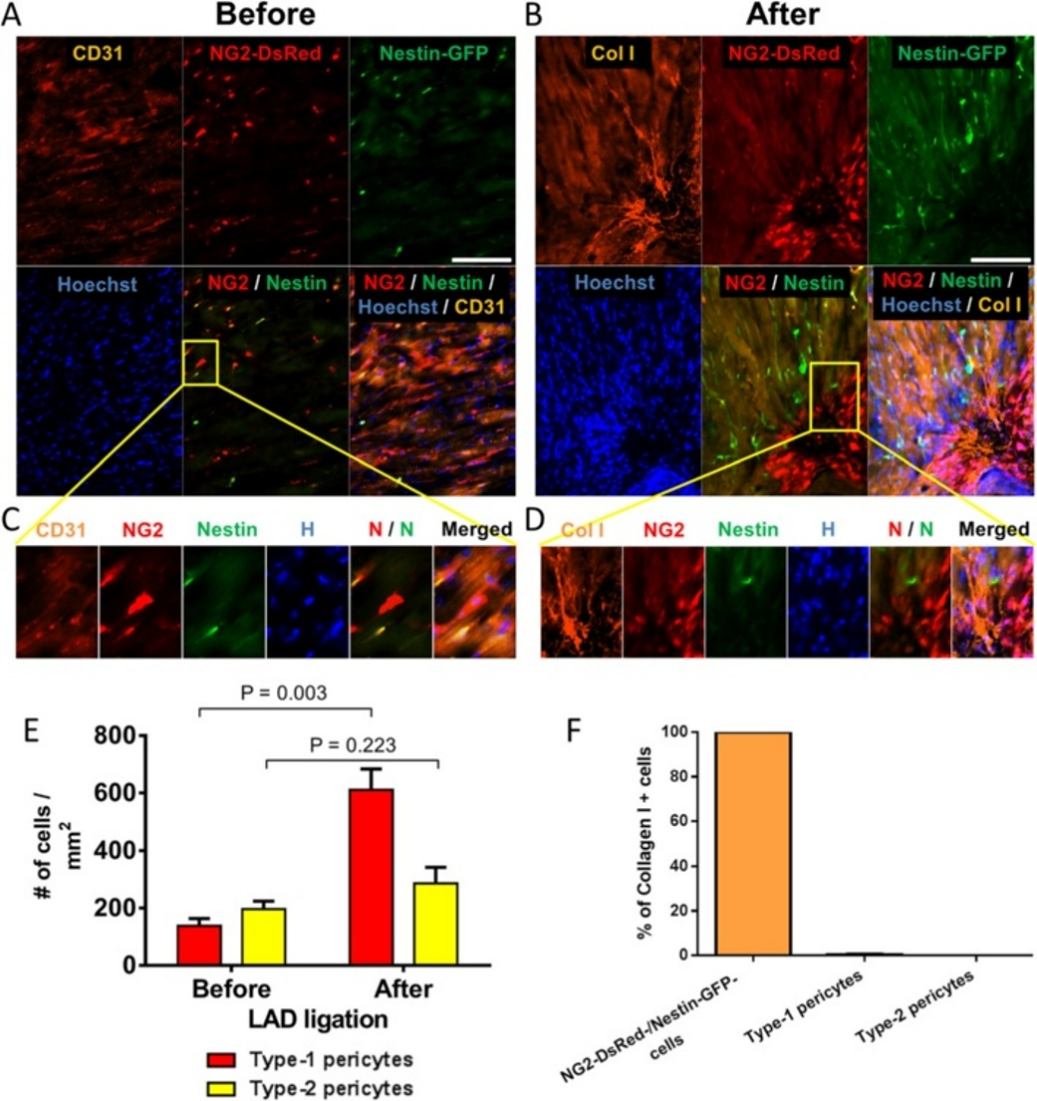
**Mouse model of myocardial infarction.** Schematic illustration of myocardial infarction (MI) in Nestin-GFP/NG2-DsRed double-transgenic mice. **(A)** A left lateral thoracotomy was performed on anesthetized and ventilated animals to expose the heart. The left anterior descending coronary artery was permanently ligated, forming a localized ischemic area. This surgical procedure mimics pathophysiological aspects of MI. **(B)** Gross anatomy representative of normal and fibrotic mouse hearts used in the study. Histology of representative control **(C)** and infarcted **(D)** hearts. Longitudinal sections of paraffin-embedded myocardial tissue were stained with hematoxylin and eosin (H&E) stain. Cardiac fibrosis was evaluated by Van Gieson’s (peach, fibrillar collagen; pink, myocardium) and Masson’s trichrome (blue, fibrillar collagen; red, myocardium) staining 2 weeks after MI.

Whether pericytes participate in postinfarct fibrosis is unknown. Ultrastructural studies determined that contractile cells in MI scars include pericytes [66], but in the absence of pericyte markers the question of pericyte contribution to cardiac fibrosis remains unanswered. We detected type-1 and type-2 pericytes in the perivascular space of CD31^+^ cardiac vessels (Figure 5A,C). To verify whether pericytes participate in fibrous tissue accumulation after myocardial injury, we induced infarction in Nestin-GFP/NG2-DsRed transgenic mice by ligating the LAD coronary artery (Figure 6A). The hearts were harvested 14 days later (Figure 6B), sectioned, and stained with hematoxylin and eosin, Van Gieson, and Masson’s Trichrome to visualize the tissue scar. Myocardial infarcts were composed of dense scar tissue characterized by increased interstitial fibrosis (Figure 6B,C,D). They were identified in all mice that underwent successful LAD ligation (Figure 6D). Sham operated control animals did not show fibrosis (Figure 6C). Fibrosis was thus related to occlusion of the LAD artery rather than any other surgical effects. Type-1 pericytes increased significantly in the infarcted area (pre injury, 138 ± 25 cells/mm^2^; post injury, 611 ± 72 cells/mm^2^; *P* = 0.003), but type-2 pericytes did not (pre injury, 196 ± 28 cells/mm^2^; post injury, 286 ± 56 cells/mm^2^; *P* = 0.223) (Figure 5B,D,E). Although type-1 pericytes concentrated in the area of the infarct, they did not produce type I collagen, which also accumulated there (Figure 5B,D,F). Collagen was produced by cells that did not express the pericytic marker NG2 proteoglycan (Figure 5B,D,F). Thus, our results imply that type-1 pericytes are recruited to the scar tissue after MI but do not contribute to tissue fibrosis.

### Type-1 pericytes participate in the scar formation after spinal cord lesion

**Figure 7:**
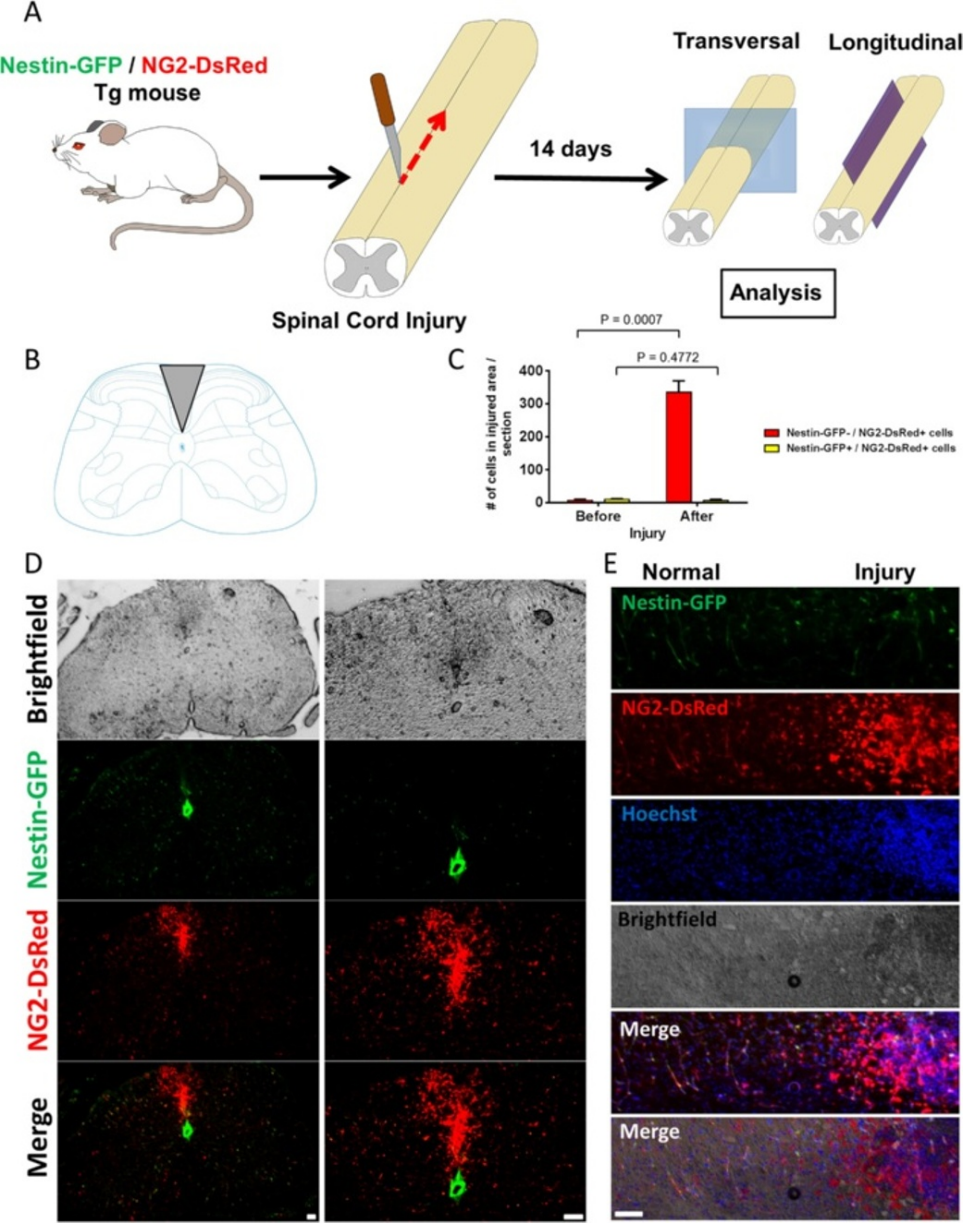
**Type-1 (Nestin-GFP** ^**–**^ **/NG2-DsRed** ^**+**^ **) pericytes accumulate after spinal cord injury** ***in vivo*** **. (A)** Spinal cord injury by dorsal funiculus incision in Nestin-GFP/NG2-DsRed mice. Spinal cord transverse and longitudinal sections were analyzed 2 weeks after injury. **(B)** Mouse spinal cord transverse-sectioned at the level of lumbar segment 5 (L5) (modified with permission from *ALLEN Spinal Cord Atlas*[72]), illustrating the area where the injury was performed (gray). **(C)** Quantification of Nestin-GFP^–^/NG2-DsRed^+^ and Nestin-GFP^+^/NG2-DsRed^+^ cells before and 14 days after injury (*n* = 3 mice, 10 spinal cord sections from each). **(D)** Photomicrographs of transverse section at L5, 14 days after injury, illustrating the distribution of Nestin-GFP^*+*^ and NG2-DsRed^*+*^ cells. Second column shows the images in the first column at higher magnification. GFP and DsRed fluorescence images are illustrated. Top panels, brightfield images; bottom panels, merged images. Note that type-1 pericytes accumulated in the tissue formed after injury, but almost no Nestin-GFP^+^/NG2-DsRed^+^ cells were detected in this area. Nestin-GFP^+^/NG2-DsRed^–^ cells, or ependymal cells, line the central canal in the spinal cord. **(E)** Photomicrographs of a longitudinal section of a spinal cord 14 days after injury. Nestin-GFP, NG2-DsRed, and their corresponding brightfield, merged fluorescence, and merged fluorescence and brightfield images are shown. Note the higher number of Nestin-GFP^–^/NG2-DsRed^+^ cells in the injured area. Scale bars = 100 μm.

PDGFRβ^+^ cells play an important role in scar formation after spinal cord injury [36], contributing the most collagen [38]. Since NG2 proteoglycan [67] and PDGFRβ [68] are expressed in pericytes, we examined whether NG2^+^ pericytes accumulate in the scar formed after spinal cord injury. We examined cell localization and the lesion’s fluorescence profile before and after performing dorsal spinal cord hemisection on Nestin-GFP/NG2-DsRed mice (Figure 7A,B). We found that type-1 pericytes increased after injury (pre injury, 7.7 ± 3.0 cells; post injury, 335 ± 34 cells), while the number of Nestin-GFP^+^/NG2-DsRed^+^ cells did not change significantly (pre injury, 10.0 ± 2.1; post injury, 7.0 ± 3.2 cells) (Figure 7C) at the injury area, the dorsal funiculus. Type-1 pericytes localized at the scar after injury, while Nestin-GFP^+^/NG2-DsRed^+^ cells distributed throughout the spinal cord section (Figure 7D,E). Note that a fraction of Nestin-GFP^+^/NG2-DsRed^+^ cells correspond to oligodendrocyte progenitors [69], which are evenly distributed in the gray and white matter in the adult spinal cord [41] but do not form the scar after injury [70]. Nestin-GFP^+^/NG2-DsRed^–^ cells correspond to ependymal cells and are only found around the ependymal canal [71].

### Type-1 pericytes differ from PDGFRβ^+^ cells and accumulate after brain injury

**Figure 8:**
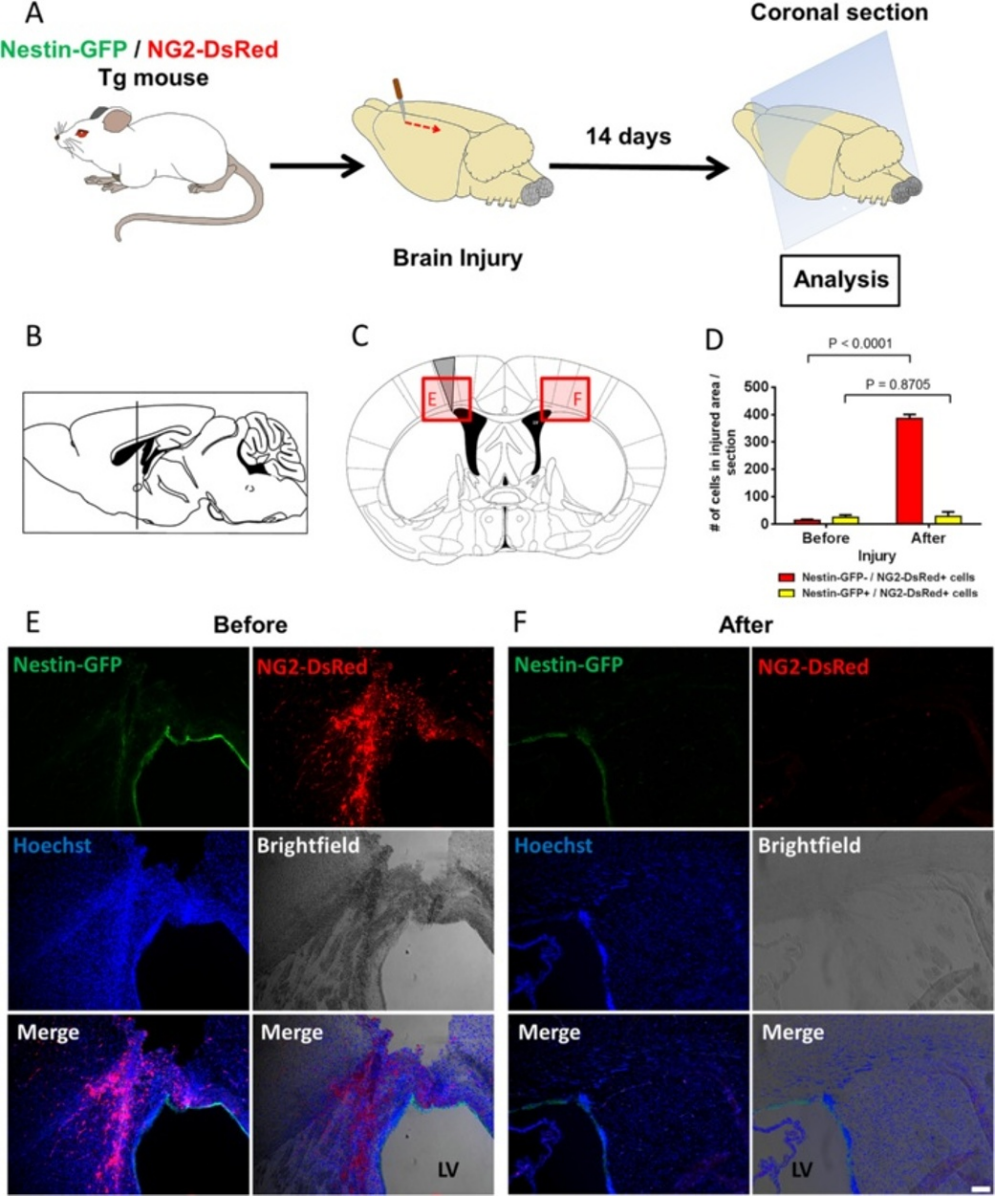
**Type-1 pericytes accumulate in the scar formed after brain injury. (A)** Experimental protocol. Brain coronal sections analyzed 2 weeks after cortical injury in Nestin-GFP/NG2-DsRed mice. **(B)** Mouse brain sagittal section (modified with permission from [75]). Vertical line indicates the site of the coronal section in (C), 0.02 mm rostral to the bregma. **(C)** Mouse brain coronal sections (modified with permission from [75]) illustrating the site of the cortical injury (gray). Shaded boxes and areas outlined in red are areas shown in (E) and (F). **(D)** Quantification of Nestin-GFP^–^/NG2-DsRed^+^ and Nestin-GFP^+^/NG2-DsRed^+^ cells before and 14 days after injury (*n* = 3 mice, 10 brain sections from each preparations). **(E)** Representative brain coronal section magnifying the cortical injury represented in (B). Note that type-1 pericytes predominate over type-2 in the scar formed after brain injury. Panels show GFP and DsRed fluorescence, brightfield, all fluorescence images merged, and all the images merged with brightfield. Nuclei were stained with Hoechst. **(F)** Representative brain coronal section magnifying the region contralateral to the injury represented in (B) in the same animal as used in (E). Note that Nestin-GFP^–^/NG2-DsRed^+^ cells developed in the scar post injury but were rarely observed in the uninjured contralateral region. Scale bar = 100 μm. LV, lateral ventricle.

**Figure 9:**
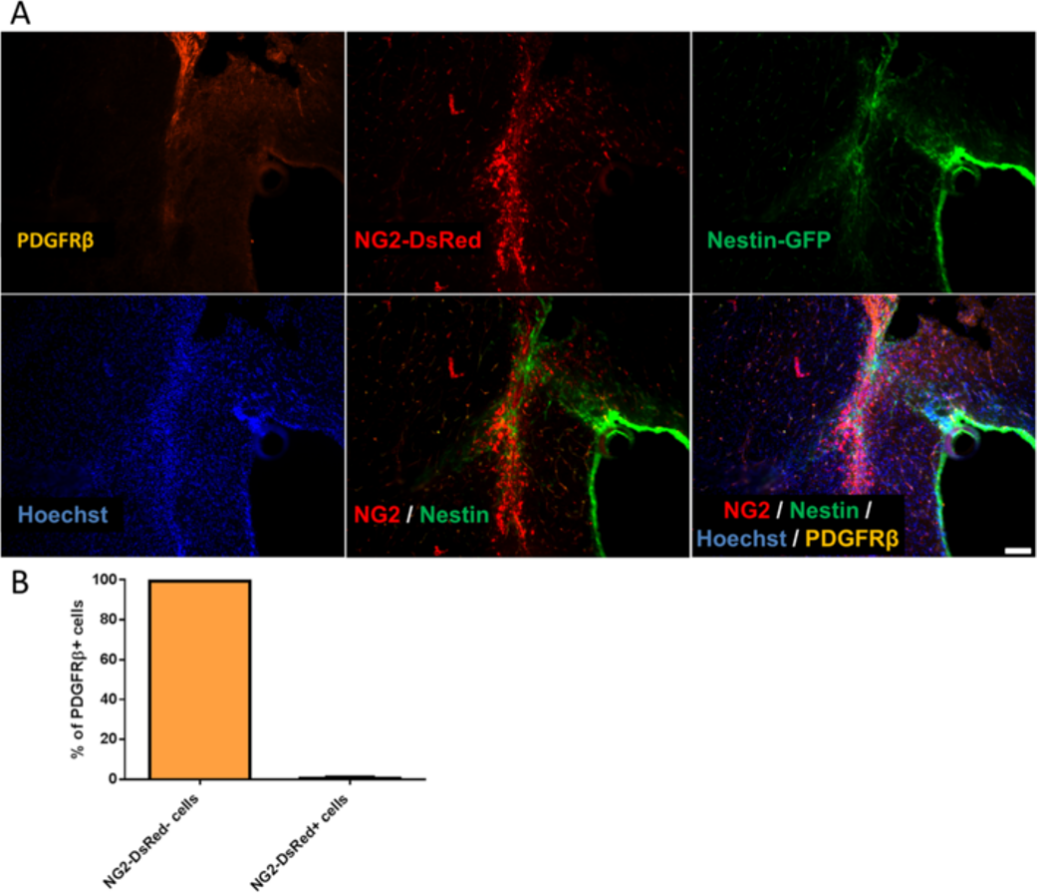
**PDGFRβ** ^**+**^ **is expressed in nonneural tissue formed after brain lesion. (A)** Representative brain coronal sections 14 days after cortical injury. All panels show identical areas with PDGFRβ staining, NG2-DsRed, Nestin-GFP^+^, Hoechst, and combined fluorescent images. Note that although type-1 pericytes accumulate in the nonneural tissue formed after brain contusion, they differ from PDGFRβ^+^ cells. **(B)** Percentage of NG2-DsRed^–^ and NG2-DsRed^+^ cells expressing PDGFRβ. Scale bar = 100 μm.

Fibrotic scars at the site of brain injury inhibit axonal regeneration [73]. Whether their cellular components are the same as those described in the spinal cord scar is not known. To determine whether type-1 pericytes are found in brain scars, we examined the location of Nestin-GFP^+^ and NG2-DsRed^+^ cells before and 14 days after injuring the brain cortex of Nestin-GFP/NG2-DsRed mice (Figure 8A,B,C). Consistent with our data on spinal cord injury (Figure 7), type-1 pericytes were observed at the injured site (387 ± 14 cells) (Figure 8D,E) but rarely in noninjured cortex (28.3 ± 17 cells) (Figure 8D,F). Nestin-GFP^+^/NG2-DsRed^–^ cells were consistently found in the subventricular zone, a neurogenic area along the walls of the brain’s lateral ventricle, where proliferating progenitor cells and quiescent neural stem cells express Nestin [69]. As in the spinal cord, Nestin-GFP^+^/NG2-DsRed^+^ cells comprise two cell populations – type-2 pericytes and oligodendrocyte progenitors – which are broadly distributed in the brain and do not participate in scar formation after injury. Surprisingly, when we stained the brain sections with PDGFRβ antibody 2 weeks after injury, we detected accumulations of both cell populations in the injured area (NG2^+^ pericytes and PDGFRβ^+^ cells), and they did not overlap (Figure 9A,B). As PDGFRβ is a known marker of other cell types, such as fibroblasts [38, 74], our results suggest that PDGFRβ^+^ cells, which contribute functionally to CNS fibrosis [36], differ from NG2^+^ pericytes. The role of type-1 pericytes in brain injury and their contribution to fibrosis require further study.

## Discussion

This study demonstrates that only the nonneurogenic and nonmyogenic type-1 pericyte, which we identified in skeletal muscle [30], proliferates and accumulates at injured sites in the lungs, kidney, heart, spinal cord, and brain. Our results also show that this pericyte subtype corresponds to a subpopulation of collagen-producing cells only in the lungs, not in the kidney and heart, indicating that its role in tissue fibrosis is organ dependent. After brain injury, type-1 pericytes differ from PDGFRβ^+^ cells at the core of the fibrous scar. Future therapies that target type-1 pericytes to improve organ recovery after injury must consider the cell and organ differences reported here.

### Two pericyte subpopulations in skeletal muscle, lung, kidney, heart, spinal cord, and brain

Pericytes have been classically subdivided in two groups based on their ontogeny: during development, most of them derive from the mesoderm [76–83], while CNS and thymus pericytes derive from the ectoderm [84–89]. Our data show that peripheral and CNS pericytes share the same markers, supporting the idea that they could share some characteristics [90, 91]. Their similarity may be explained by cell reprogramming from ectodermal to mesodermal during development [92–94]. However, our results indicate that pericyte subtypes do not play the same roles in different tissues.

In the present work, we detected the presence of both type-1 and type-2 pericytes in tissues derived from different germ layers [90]. How pericyte subpopulations are related by lineage and function is the subject of current investigation by several groups. Whether both pericyte subtypes derive from the same progenitor by asymmetric division or distinct developmental stages is an important question that remains open. At the adult stage, our previous data suggest that pericyte subtypes do not interconvert, as they differ significantly in their functions when exposed to the same physiological/pathological microenvironments [30, 31, 34, 95].

Although we found the two subtypes in several tissues, we do not know how type-2 pericytes (Nestin-GFP^–^) respond to pathological conditions. All of the injury models studied here increase scar tissue, which seems to involve only type-1 pericytes. Determining the role of type-2 pericytes in these tissues and whether they are affected by other pathological conditions will require testing or developing other injury models.

Future work should also explore why type-1 pericytes accumulate in several tissues after injury, because in most cases they do not seem to produce extracellular matrix collagen.

### Pericyte participation in peripheral organ repair after injury

Normal tissue homeostasis, regeneration, and repair rely on resident stem cells. In mammals, incomplete regeneration has been attributed to an insufficient number or malfunction of stem cells and a rapid fibroproliferative response after wounding. Excessive scar formation can lead to organ failure. Various cell types, including pericytes, have been implicated in fibrous tissue formation in several organs [57], but whether pericyte participation varies between organs and only a specific subtype contributes to scarring remains unexplored.

Pericytes have been shown to differentiate into collagen-producing cells in models of dermal scarring [96]. In a recent lineage-tracing report, pericytes were found to contribute to formation of fibrous tissue after injury to skeletal muscle and dermis [22]. Another recent report demonstrates that liver pericytes, also known as hepatic stellate cells, are the major source of collagen during chronic hepatic disease [20].

In contrast to the dominant role of pericytes in skin, skeletal muscle, and liver fibrosis [20, 22], their contribution to fibrous tissue formation in other organs remains controversial. Some kidney and lung studies show important participation [21, 65]; other studies do not [23, 97]. Possible explanations include the use of different mouse models, the small percentage of cells undergoing recombination in some of them, and pericyte markers expressed by other cells, such as fibroblasts. Our study confirms that type-1 pericytes accumulate in the injured area in several organs, although we detected a fibrogenic role only in the lungs.

We used single time points to assess the effects of injury on tissue fibrosis based on the literature [21, 23, 35, 65, 97]. A longitudinal rather than cross-sectional study may clarify the relationship between pericyte subtypes, tissue inflammation and fibrosis stages. Additionally, the repair of each organ may depend on the nature of the injury (ischemic or chemically induced). These questions should be examined in future works.

In a recent report, we showed in skeletal muscle that only the subset of type-1 pericytes not involved in myogenesis produces collagen, thus contributing to fibrous tissue deposition in older mice [34]. Here, we show that type-1 pericytes correspond to approximately 10% of collagen-producing cells after lung injury. In contrast, although they concentrate in the injured area of the kidney and heart, they do not produce collagen.

Our results show that type-2 pericytes are not involved in collagen production nor found at higher percentage near the scar area of the organs studied. Whether this is due to lack of response to certain signals (for example, certain receptors) remains to be elucidated. This information will help to develop drugs to block only one pericyte subtype, while preserving the beneficial roles of the other [31, 95].

### Nonneural cell participation in the central nervous system scar after trauma

Genetically altered mice provide a powerful tool for elucidating the complex cellular and molecular mechanisms underlying CNS injury, which leads to scar formation at the lesion site. Various nonneural cells play critical roles. Endothelial cells are activated to form new vessels [98, 99], leukocytes [100] can contribute to tissue repair, while macrophages [101] and microglia cells clear debris and recruit other cells [102–106].

Recent studies claim that most scar cells in the injured spinal cord are not glial cells. PDGFRβ^+^ pericytes have been reported to form fibrous tissue after spinal cord injury, and their genetic abrogation in the lesion results in its failure to close [36]. Goritz and colleagues claim that PDGFRβ^+^ cells are pericytes, although this marker is also present on other cells. In another recent study, the use of transgenic approaches demonstrated that PDGFRβ^+^ cells in the fibrotic core of the scar produce type I collagen and correspond to fibroblasts [38].

Here, we show that 2 weeks after spinal cord and brain injury, type-1 pericytes but not type-2 pericytes increase and accumulate at the injured site. We also show that type-1 pericytes differ from PDGFRβ^+^ cells in the injured cortex, suggesting that their role in tissue repair after CNS injury is distinct from the collagen production described for PDGFRβ^+^ cells [36, 38].

### Signaling involved in type-1 pericyte accumulation merits further research

Proliferation and migration of type-1 pericytes may be important in the pathogenesis of organ fibrosis. Several studies have shown that fibrosis activates cell division and migration [107], In particular, excessive PDGFβ activity has been associated with several human disorders, including organ fibrosis [108], and its signaling is critical for pericyte expansion and migration [109]. Future studies should focus on these signaling pathways.

Collagen and transforming growth factor beta are upregulated in tissue fibrosis [110]. Transforming growth factor beta has long been considered the most important extracellular matrix regulator [111], and also regulates cell division and migration [112]. Transforming growth factor beta and type I collagen thus facilitate the further migration of pericytes into diseased areas [107]. Additionally, pericytes express NG2 proteoglycan [113], which is very effective as a receptor, anchoring collagen to the cell surface [114, 115]. The functional significance of this NG2/collagen interaction is suggested by an increased ability of NG2^+^ cells to migrate in response to collagen [116]. Collagen accumulation may also activate the NG2 receptor and promote a cellular mitogenic response [117].

The tissue-specific variations we found in pericyte participation in the fibrotic response in different organs raise two questions: are different signaling pathways involved; and why do type-1, but not type-2, pericytes respond to the injury models studied here? These problems will be investigated.

### Need for specific pericyte markers

Here, we used NG2 and Nestin expression to detect pericyte subtypes, but we lack a single, specific, positive marker to track cell fate using recombination-based technology. We have yet to discover a membrane protein expressed in pericyte subpopulations throughout the body that would facilitate their isolation and manipulation under normal and pathological conditions.

To detect such specific markers, our future studies will screen for both pericyte subtypes isolated from Nestin-GFP/NG2-DsRed mice by microarray analysis, enabling the use of mouse genetic engineering to ablate cells. For instance, using pericyte subtype-specific marker CreER/DTA mice, as with other cell types [118, 119], might clarify their physiologic response to tissue injury.

### Distinguishing type-1 pericytes from type-2 pericytes is essential to exploiting their therapeutic potential

Our earlier work found that under optimized culture conditions only type-2 pericytes can generate neural cells [30], and transplantation studies indicate that type-2 pericytes participate in muscle regeneration, while type-1 pericytes contribute to adipose and fibrous tissue accumulation in the skeletal muscle [31, 34]. Here, we show that type-1, but not type-2, pericytes accumulate at an injured site. Whether each pericyte subset has distinct differentiation programs, self-renewal capacities, and requirements for proliferation in other tissues is unknown.

Pericytes are multipotent stem cells [4–17, 120]. Besides their role in tissue repair, some studies suggest that pericytes have important functions in several diseases [121]. Our studies support the idea that the capabilities of the pericyte subtypes we discovered differ [30]. We suggest that a pericyte subtype can be a source of easily accessible, autologous cells that can be expanded for tissue engineering and regenerative medicine. Future studies should reveal and clarify more differences in the roles of pericyte subtypes, so they can be used as cellular targets susceptible to signaling and pharmacological manipulation.

### Limitations of this study

The only marker we found differentially expressed in pericytes is Nestin-GFP, which is also expressed in other cells [122–144]. Nevertheless, this marker is not expressed in type-1 pericytes; thus, the combination of Nestin-GFP and NG2-DsRed expression allowed us to identify pericyte subpopulations [30]. We used knockin mice (Nestin-GFP and NG2-DsRed mice) in this study, to detect the collagen-producing cells at specific time points.

Although there is no consensus [23, 38, 97], traceable recombinase (Cre) systems (including NG2-Cre) revealed that pericytes represent a source of myofibroblasts in some organs and play an important role in tissue fibrosis after injury [21, 36, 57]. The knockin system used in this work identifies cells producing collagen only when they actively express NG2. We therefore cannot rule out the possibility that some pericytes lose NG2 expression in their conversion to myofibroblasts in the fibrotic tissue. Using an NG2-Cre mouse in the future may clarify this point. Although inducible Cre systems are available to trace pericytes as a whole population, tracing pericyte subtypes’ fate will require the discovery of new markers expressed in a pericyte subpopulation. Future studies should define these novel markers.

Neuron-glial 2 chondroitin sulphate proteoglycan [67, 113] is the most commonly accepted pericyte marker. Both type-1 and type-2 pericytes also express PDGFRβ [31, 34, 68] and CD146 [13, 31, 34, 145]. Although alkaline phosphatase is expressed in pericytes, it was also found in endothelial cells [9]. Whether alkaline phosphatase overlaps with one or both pericyte subtypes remains to be elucidated. In this study, we analyzed NG2^+^ pericytes; whether NG2^–^ pericytes participate in tissue fibrosis [146] remains to be analyzed. In peripheral tissues, we only detected NG2 expression in pericytes closely attached to endothelial cells. In the CNS, NG2 proteoglycan is also expressed in oligodendrocyte progenitors near pericytes; however, they are not recruited at the fibrous scar tissue after injury [70].

## Conclusions

Pericyte subpopulations respond differentially to tissue injury, and the production of collagen by type-1 pericytes is organ dependent. Future studies of pericyte subtypes functions in peripheral tissues may reveal ways to improve tissue repair under pathological conditions or with aging.

## Electronic supplementary material

Additional file 1: Figure S1: (Related to Figure 4) showing that type-1 pericytes accumulate, but do not overlap, with collagen-producing cells, in a mouse model of kidney fibrosis. Representative immunofluorescence image of an obstructed kidney section 14 days after UUO in a Nestin-GFP/NG2-DsRed mouse. All panels show the same area for different channels (collagen type I (Col I), NG2-DsRed, Nestin-GFP, Hoechst, brightfield, and merged images). Scale bar = 100 μm. (TIFF 2 MB)

## Abbreviations

CNS: central nervous system
Cre: recombinase
CreER: recombinase fused to a mutated ligand-binding domain of the human estrogen receptor
DTA: diphtheria toxin fragment A
GFP: green fluorescent protein
LAD: left anterior descending
MI: myocardial infarction
NG2: neuron-glial antigen 2
PBS: phosphate-buffered saline
PDGFRβ: platelet-derived growth factor receptor beta
UUO: unilateral ureteral obstruction.
