# Supplementary figures and images for: Type-1 pericytes accumulate after tissue injury and produce collagen in an organ-dependent manner
Source: Stem Cell Res Ther. 2014 Nov 6;5:122. doi: 10.1186/scrt512 (PMC4445991; doi:10.1186/scrt512)

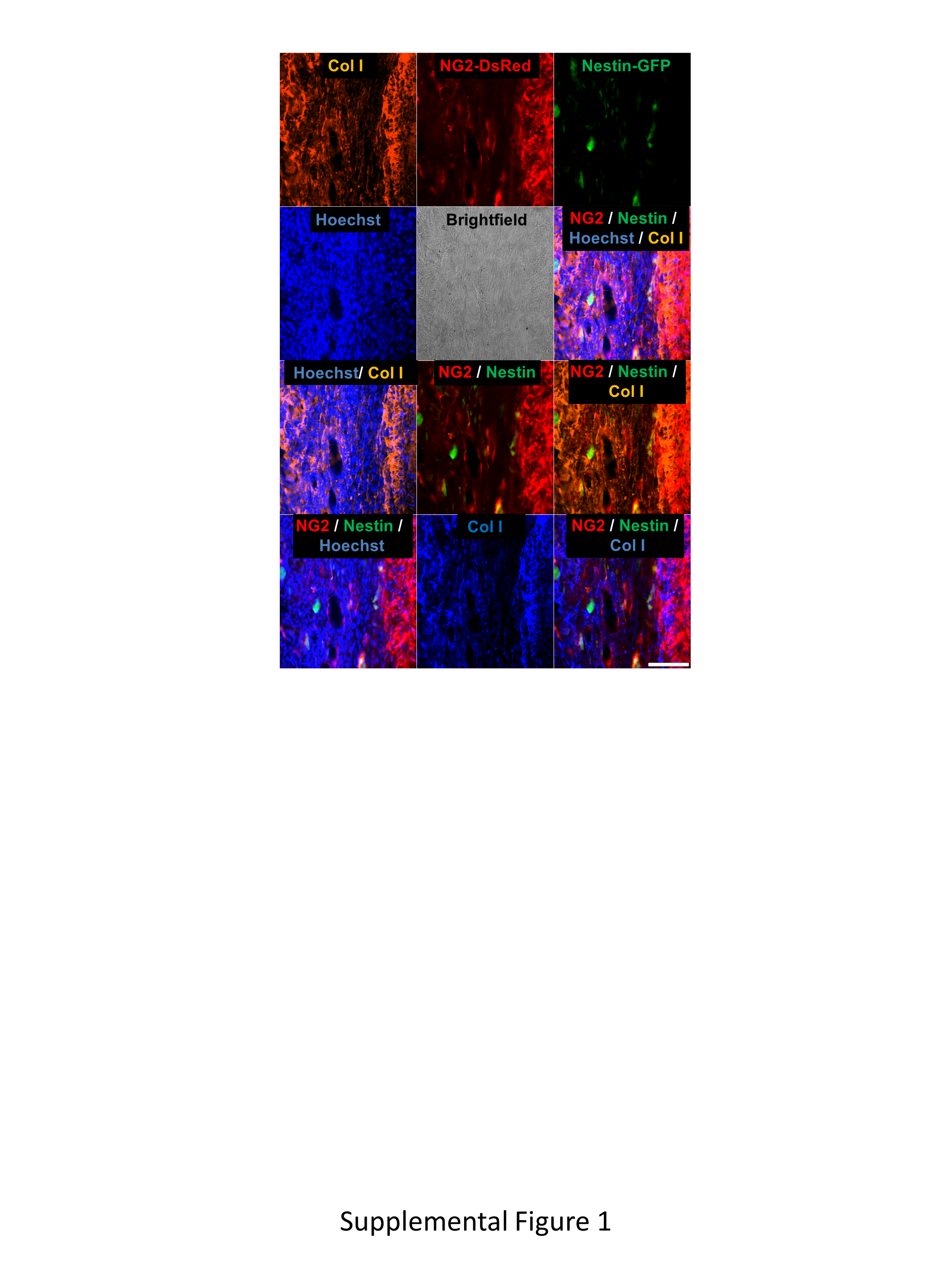

Supplement: Supplementary file 1 — Additional file 1: Figure S1: (Related to Figure 4) showing that type-1 pericytes accumulate, but do not overlap, with collagen-producing cells, in a mouse model of kidney fibrosis. Representative immunofluorescence image of an obstructed kidney section 14 days after UUO in a Nestin-GFP/NG2-DsRed mouse. All panels show the same area for different channels (collagen type I (Col I), NG2-DsRed, Nestin-GFP, Hoechst, brightfield, and merged images). Scale bar = 100 μm. (TIFF 2 MB) [file 13287_2014_434_MOESM1_ESM.tiff]
